# Self-inflicted penetrating eye injuries using a razor blade: Case report

**DOI:** 10.1186/1471-2415-9-14

**Published:** 2009-12-10

**Authors:** Hessom Razavi, Nicholas Price

**Affiliations:** 1 International Centre for Eye Health, London School of Hygiene and Tropical Medicine, London, UK; 2 Ophthalmology department, Cheltenham General Hospital, Cheltenham, UK

## Abstract

**Background:**

A 23 year old white male with a history of social and behavioural problems attempted to blind himself chemically, with alcohol, and mechanically, with a razor blade.

**Methods:**

Observational case report of a patient who self-inflicted bilateral scleral lacerations with a razor blade, after losing his job.

**Results:**

The patient sustained bilateral inferior scleral perforations, with hypotony and a right traumatic cataract. He received urgent surgical repair, and prophylactic antibiotics. There were no retinal breaks or detachments. He later underwent successful cataract surgery to the right eye.

**Conclusion:**

Self-inflicted ocular injury may be possible in non-psychotic patients, as a situational response to a life event. Urgent repair can completely restore vision in some cases. Referral for psychiatric counseling is mandatory.

## Background

Deliberate self-inflicted eye injuries are an uncommon, but serious form of self-harm [1], due to the potential for visual loss and blindness. They occur most commonly in young adults with acute or chronic psychoses [2,3]. There are psychiatric and biochemical theories for the pathogenesis [4]. Psychodynamic theories explain self-inflicted eye injuries as a form of self-mutilation, commonly associated with altered body image, and religious or sexual ideation. Disorders of neurotransmitters, particularly serotonin, have been implicated in self-harming behavior in a number of conditions, including Prader-Willi syndrome and Lesch-Nyhan syndrome. There are also many references to self-inflicted eye injuries in mythology and ancient literature, the most well known of which may be the story of Oedipus [5].

Many mechanisms have been cited for self-inflicted eye injury, including use of the fingers in self-enucleation, blunt and sharp instruments such as pens and knives, head banging and mechanical scratching of ocular surfaces [4]. Whatever the cause and mechanism, successful self-inflicted injury to the eye often brings the patient relief from anxiety [3].

## Case presentation

A 23 year old man was referred from the emergency department with sudden bilateral eye pain and visual loss. On further history, he had been made redundant at work that day, and had proceeded to 'try and blind' himself as a result. He had first poured alcohol over eyes, which was unsuccessful. He had then super-glued his eyelids open, cut both eyes with a razor blade, and pressed on his eyes. He denied consuming any alcohol or illicit drugs prior to the event. He also denied hallucinations and suicidal ideation. Past medical history included asthma, failure to thrive, and a number of behavioural problems including depression, nocturnal enuresis, and being bullied at school. There was no past history of self harm. He had previously taken Amitriptyline as a teenager, which had since been ceased.

On examination, he was a disheveled young man with blood stains on his face. He had a blunted affect but was otherwise coherent and appropriate. Visual acuity (VA) was hand movements (HM) in the right eye (OD) and count fingers (CF) in the left eye (OS). On slit-lamp biomicroscopy, both eyes were soft, with inferiorly peaked pupils. There was an inferior sub-conjunctival haemorrhage OD, with an underlying scleral laceration. There was an inferior conjunctival bleb OS, filled with visible uvea. Corneal folds, superficial punctate keratopathy and shallow anterior chambers were noted in both eyes. A superior cataract was noted in the OD. The retinas could not be assessed. The patient was given oral levofloxacin 500 mg, and arrangements were made for urgent surgical repair. Preliminary psychiatric assessment of the patient elicited a sense of regret at having attempted to blind himself, as well as chronic indicators of low mood, self esteem and confidence.

Operative repair was carried out 4 hours later by a consultant ophthalmologist, under general anaesthesia. Horizontal perforating scleral lacerations, measuring approximately 1.1 mm OD and 1.3 mm OS in length, were found approximately 3 mm inferior to the limbus in both eyes. The prolapsed uvea OS was repositioned into an intraocular position, after which the scleral lacerations and conjunctiva were repaired with 8.0 nylon and vicryl sutures, respectively. Subconjunctival cefuroxime and gentamicin were given. Indirect ophthalmoscopy revealed a flat retina OS, and poor view OD due to the cataract. The patient was awakened from general anaesthesia without difficulty.

The following day the VA was CF OD, and 6/36 OS, and B-scan ultrasonography showed flat retinas in both eyes, with localized inferior vitreous haze/blood. Over the remainder of the five day admission, VA progressively improved to 6/12 OS, but remained worse OD due to the cataract. Further psychiatric assessment elicited no psychosis or suicidal thoughts, poor mood and insight, and a tendency towards social anxiety and avoidance. The psychiatric plan was for discharge when medically fit, and follow up was arranged. The patient was subsequently commenced on Fluoxetine 20 mg daily, which was ceased after 12 months by the psychiatric care team.

Over the two months after discharge the patient attended regular ophthalmic outpatient follow up. VA OS continued to improve to 6/5+4, but remained stable OD at HM. The right cataract progressed to the point where no view of the right retina was possible. Uncomplicated cataract surgery was carried out OD, 81 days after the initial injury. A month later VA OD was 6/6+3 with pin-hole correction, and the patient was reading well. Nine months after the injury, VA with both eyes was 6/5 after refraction. Early posterior capsular opacification was noted OD, and an early nuclear sclerotic cataract noted OS, presumably a late developing complication of the initial trauma. Treatment of these findings, as well as refraction for the lack of accommodation in the pseudophakic eye, was discussed and further follow up arranged. However the patient failed to attend and was lost to follow up. There has been no reported repetition of deliberate self-harm since this episode.

## Conclusion

Penetrating injuries with needles have previously been observed in several cases [6,7]. This patient used super-glue, razor blades and digital pressure to deliberately self-inflict penetrating eye injuries; a mechanism not previously cited in the literature, to the author's knowledge. Urgent surgical repair, antibiotic prophylaxis, and timely cataract surgery at a later date were successful in restoring vision. The case highlights the possibility of self-inflicted ocular injury in non-psychotic patients, as a situational response to a life event.

However, studies of self-harmers who present to hospitals that used standard diagnostic criteria have shown that more than 90% of these people had at least one psychiatric disorder, most commonly depression, followed by substance abuse and anxiety disorders [8]. In this patient there were clues to a previous mood disorder, and chronic indicators of low mood at presentation. Furthermore, the possibility of psycho-active drug abuse by this patient cannot be excluded on history taking alone. The authors can therefore not rule out the possibility of an acute psychotic episode in this patient, due to an undetected psychiatric disorder, and/or psycho-active drug abuse.

Psychiatric referral for self-harm is mandatory, due to the possibility of repeat episodes and the increased risk of suicide; up to 25% of people with self-inflicted harm repeat the self-harm within 1 year of the first episode [9], and even more will repeat without presenting [10]. At least 5% of people seen at a hospital after self-harm will have committed suicide within 9 years [11]. There is presently no published evidence on the likelihood of repetition of self-inflicted eye injuries, which could reflect the difficulties in undertaking research into such rare events. However, this could provide a direction for future research.

## Consent

Written informed consent was obtained from the patient for publication of this case report and any accompanying images. A copy of the written consent is available for review by the Editor-in-Chief of this journal.

## Competing interests

The authors declare that they have no competing interests.

## Authors' contributions

HR contributed to clinical management of the patient, collection of clinical information on the patient, literature search, design of the case report and writing of the article. NP contributed to patient management (surgery), documentation of clinical information, design of the case report and revision of the manuscript. Both authors have read and approve the final manuscript.

## Pre-publication history

The pre-publication history for this paper can be accessed here:

http://www.biomedcentral.com/1471-2415/9/14/prepub
